# E-cigarette or vaping product use–associated lung injury and state-level cannabis policies

**DOI:** 10.1186/s42238-020-00053-x

**Published:** 2020-12-31

**Authors:** Danielle M. Smith, Maciej L. Goniewicz

**Affiliations:** Department of Health Behavior, Roswell Park Comprehensive Cancer Center, Elm and Carlton Streets, Buffalo, NY USA

**Keywords:** E-cigarette or vaping product use–associated lung injury, Vaping, Cannabis, Tocopherol acetate, Policy, Legalization

## Abstract

**Background:**

Multiple cases of e-cigarette or vaping product use–associated lung injury (EVALI) have been reported in the USA, which have been attributed to informally obtained cannabis oil vaping devices. This report estimated whether cumulative incidence of EVALI differed according to state-level cannabis policy.

**Methods:**

Publicly available information was used to estimate the cumulative incidence of EVALI. Odds of incident EVALI were calculated according to policy status (active, legal adult-use recreational policy vs. no legal access). Figures were statistically compared using chi-square tests.

**Results:**

Estimated cumulative incidence of EVALI was 5.19 per 100,000 cannabis users across all states with recreational cannabis policies (95% CI 4.70–5.72), and 15.89 per 100,000 cannabis users across all states with no legal access to cannabis (95% CI 14.88–16.96). Odds of EVALI were 3.06 times higher (95% CI 2.71–3.45) among users living in states with no legal access to cannabis compared to users in states with active recreational policies, with significant differences detected according to policy exposure (*χ*^2^ = 385.57, *p* < 0.0001).

**Conclusions:**

Estimates suggest there may have been a protective effect of state-level, recreational adult-use cannabis policies on incident EVALI. Effects of specific state-level regulations (e.g., laboratory testing, product quality standards, tracking systems) should be considered alongside additional geographic indices in future assessments of EVALI causes.

## Introduction

In late 2019 and early 2020, numerous cases of e-cigarette or vaping product use–associated lung injury (EVALI) were reported in the United States (CDC 2020). As of February 18, 2020, the US Centers for Disease Control and Prevention (CDC) documented 2807 cases of EVALI, including 68 fatalities (CDC 2020). Multiple sources have implicated tocopherol (vitamin E) acetate, a vaping product additive found in many illegally obtained products, as a significant contributor to this outbreak (CDC 2020; Blount et al. 2019a; FDA 2019; Blount et al. 2019b). Available information suggests the majority of EVALI patients reported vaping illegally or informally obtained cannabis-derived oils (CDC 2020). Additionally, the CDC notes that “Evidence is not sufficient to rule out the contribution of other chemicals of concern, including chemicals in either THC of non-THC products, in some of the reported EVALI cases.” (CDC 2020). During the outbreak, multiple statements from the US government health agencies and professional medical organizations advised the public to discontinue vaping of any kind (CDC 2020; FDA 2019). The CDC and FDA recommended that “…people not use THC-containing e-cigarette, or vaping, products, particularly from informal sources like friends, family, or in-person or online dealers.” (CDC 2020).

In line with growing global trends (Hall et al. 2019), several US states have liberalized their policy approaches to cannabis. While cannabis remains illegal at the federal level in the USA due to its designation as a Schedule 1 substance (National Academies of Science Engineering and Medicine 2017), 33 US states have some form of legal cannabis policy, including 11 states that permit legal adult-use for recreational purposes. There are a number of circulating arguments that either favor or oppose continued cannabis liberalization, including issues related to health, safety, morality, and social justice (McGinty et al. 2017). Irrespective of these viewpoints, there are important differences in state-level cannabis policies to consider in the context of potential contributors to EVALI. States that have legalized recreational cannabis for adult-use frequently implement testing and quality control measures to assure users’ safety, including for cannabis formulations used in vaping devices (Leafly 2019). The absence of any state regulatory oversight in areas where recreational cannabis use remains illegal may have placed cannabis users who live in those states at elevated risk for EVALI. To investigate this issue, we sought to estimate the cumulative incidence of EVALI according to state-level cannabis policy status.

## Methods

We ascertained state-level data on confirmed or probable cases of EVALI from publicly available sources (state health department websites and press releases) reported in all 50 states between September 2019 and March 2020. Obtained case counts were checked against categorical information provided by CDC as of February 18, 2020 (CDC 2020); the concordance rate was 78%. Next, we obtained state-level prevalence and population estimates of past month cannabis users aged 12 and older using the most recent publically available data from the National Survey on Drug Use and Health (NSDUH, 2017–2018) (U.S. Department of Health and Human Services 2019). We focused on figures for past month users due to potentially heightened exposure to chemicals associated with EVALI that may stem from greater frequency of product use (Goniewicz et al. 2018). We calculated and compared the incidence of EVALI among past month cannabis users in states that had legal adult-use recreational marketplaces permitting retail sales of cannabis products at the beginning of the epidemic (March 2019) (Alaska, California, Colorado, Massachusetts, Nevada, Oregon, Washington) to states where THC-containing cannabis products remain illegal (i.e., no legal recreational/medicinal cannabis use or CBD-only policy; Alabama, Georgia, Idaho, Indiana, Iowa, Kansas, Kentucky, South Carolina, South Dakota, Tennessee, Texas, Virginia, Wisconsin, Wyoming) (Marijuana Policy Project 2020). We conducted sensitivity analyses excluding the state of Massachusetts from our calculations due to the enactment of a ban on vaping products during the outbreak (Lannan n.d.), which may have impacted users’ risk of exposure to chemicals of concern in EVALI. We also excluded states with medical-only use policies from our analysis in order to facilitate a direct comparison of EVALI case rates on the basis of legal status (i.e., complete state legality versus no legality at all). States that had current or pending recreational use status but did not have active cannabis retail markets (e.g., Vermont, Illinois) were excluded from analyses, since users in these areas would have been unable to acquire legal, regulated vaping products at the time preceding or during the outbreak. We calculated the cumulative incidence and odds of EVALI according to state-level cannabis policy status, and assessed between-group differences using a chi-square test. To examine the possible impact of state population size on estimates, we used state-level estimates from the 2018 American Community Survey (United States Census Bureau 2018) to perform Pearson correlation analyses to examine associations between cumulative incidence rates of EVALI, population size, and state-level prevalence of cannabis use. Linear regression was used to examine the association between cumulative incidence of EVALI and state-level cannabis policy, adjusting for population size. Stata *v*.16.0 was used for all analyses.

## Results

Estimates as of March 2020 suggest there were 2958 confirmed or probable cases of EVALI across all 50 states, including 402 cases among an estimated 7,748,000 past month cannabis users who lived in states with active recreational cannabis sales markets, and 897 cases among an estimated 5,645,000 past month cannabis users who lived in states with no legal access to cannabis. Using these figures, the cumulative incidence of EVALI was 5.19 per 100,000 past month cannabis users across all states with recreational cannabis use policies (95% CI 4.70–5.72), and 15.89 per 100,000 past month cannabis users across all states with no legal access to cannabis (95% CI 14.88–16.96). State-specific estimates can be viewed in Table 1. The estimated odds of developing EVALI were 3.06 times higher (95% CI 2.71–3.45) among past month cannabis users living in states with no legal access to cannabis compared to past month cannabis users in states with active recreational use policies, with significant differences detected according to policy exposure (*χ*^2^ = 385.57, *p* < 0.0001). When excluding Massachusetts, cumulative incidence of EVALI was 3.96 cases per 100,000 past month cannabis users across all states with recreational cannabis use policies (95% CI 3.52–4.46). This exclusion resulted in 4.01-fold greater odds (95% CI 3.49–4.60) of developing EVALI being in states with no legal policy compared to locations that had active recreational use policies in place (*χ*^2^ = 475.74, *p* < 0.0001).

**Table 1:** State-specific estimated cumulative incidence of e-cigarette or vaping product use-associated lung injury (EVALI), by policy status

State	Estimated EVALI cases	Estimated no. past month cannabis users^a^	EVALI cases per 100,000 past month cannabis users (95% confidence interval)
**States with recreationally legal cannabis use**			
Alaska	1	97,000	1.03 (0.18–5.84)
California	210	3,955,000	5.31 (4.63–6.08)
Colorado	8	819,000	0.98 (0.49–1.93)
Massachusetts^b^	127	806,000	15.76 (13.24–18.75)
Nevada	6	379,000	1.58 (0.72–3.45)
Oregon	23	668,000	3.44 (2.29–5.17)
Washington	27	1,024,000	2.64 (1.8–3.84)
Total	402	7,748,000	5.19 (4.70–5.72)
**States where cannabis is illegal/CBD-only use**			
Alabama	16	339,000	4.72 (2.90–7.67)
Georgia	42	710,000	5.92 (4.37–8.00)
Idaho	11	117,000	9.40 (5.24–16.84)
Indiana	128	567,000	22.57 (18.98–26.84)
Iowa	60	185,000	32.43 (25.19–41.74)
Kansas	24	151,000	15.89 (10.68–23.65)
Kentucky	22	304,000	7.24 (4.77–10.96)
South Carolina	40	352,000	11.36 (8.34–15.47)
South Dakota	13	51,000	25.49 (14.89–43.61)
Tennessee	78	482,000	16.18 (12.96–20.19)
Texas	250	1,402,000	17.83 (15.75–20.18)
Virginia	103	512,000	20.12 (16.58–24.39)
Wisconsin	108	436,000	24.77 (20.51–29.90)
Wyoming	2	37,000	5.41 (1.48–19.71)
Total	897	5,645,000	15.89 (14.88–16.96)

^a^Estimates were derived from state health department websites and press releases, the US Centers for Disease Control and Prevention (CDC), and past year and past month estimates of cannabis use from the 2017–2018 National Survey on Drug Use and Health (NSDUH)

^b^Odds of EVALI were 3.06 higher (95% CI 2.71–3.45) in states with no legal policy (*χ*^2^ = 385.57, *p* < 0.0001). When removing Massachusetts from the analysis, there were 3.96 cases per 100,000 past month cannabis users in legalized states; this exclusion resulted in the odds of EVALI being 4.01-fold higher(95% CI 3.49–4.60) in states with no legal policy compared to locations that had legal markets in place (*χ*^2^ = 475.74, *p* < 0.0001)

Correlation analyses revealed no association between cumulative incidence of EVALI per 100,000 cannabis users and state-level population (*r*^2^ = − 0.04, *p* = 0.858); population size was not associated with prevalence of cannabis use (*r*^2^ = − 0.06, *p* = 0.801), and, on average, did not differ according to cannabis policy status (*t*(19) = − 0.753, *p* = 0.461). Cumulative incidence of EVALI per 100,000 cannabis users was significantly and negatively correlated with prevalence of use, such that increasing prevalence of state-level cannabis use was associated with declines in cumulative incidence of EVALI (*r*^2^ = − 0.612, *p* < 0.05). Results from a linear regression controlling for state-level population size reinforced our initial findings noting significantly fewer cases among cannabis users in states with legalized cannabis policies relative to areas with no legal policies in place (*B* = − .0001147, 95% CI − .0001933 to − .0000361, *p* = 0.007).

## Discussion

Our calculations indicate that the cumulative incidence of EVALI was approximately three-fold greater among cannabis users in states with no legal access to cannabis versus cannabis users that reside in areas with legal adult-use cannabis marketplaces. This may have been due to legal access to regulated and/or tested products. These findings suggest that differences in state-level cannabis policies should be considered in future investigations into the causes of EVALI.

The EVALI outbreak has largely been attributed to the use of informally/illegally obtained cannabis oil vaping devices (CDC 2020; Blount et al. 2019b), and our findings add to existing evidence suggesting incident EVALI was higher in areas without legal access to cannabis (Wing et al. 2020). In areas with legal adult-use cannabis marketplaces, it is possible that more cannabis users obtained their vaping products through licensed retail channels, which may be less prone to selling potentially harmful products containing agents such as vitamin E acetate (Wing et al. 2020; Duffy et al. 2020; Taylor et al. 2019). While more research is needed on the health effects of constituents used in all vaping products (Werner et al. 2020; National Academies of Science Engineering and Medicine 2018), we speculate that legal environments may have offered potential for implementing product quality standards and other requirements determined by state regulatory bodies. In turn, this may have provided protection for consumers who purchased cannabis products through legal channels. Regulatory authorities may also impose fines or legal sanctions on producers and/or retailers found to be violating state-determined product protocols, which may act as a deterrent toward the use of additives such as vitamin E acetate in their products. While our analysis did not examine specific policy elements that may have mitigated the outbreak or exerted protective effects, we speculate that several may be at play. For instance, product standards and laboratory testing requirements for products authorized for retail sale may provide a layer of protection in preventing the addition of problematic constituents, and identifying problematic constituents or batches of product prior to the point-of-sale. Additionally, the existence of “seed to sale” tracking and inventory systems may be used to identify products sold through dispensaries that are subsequently found to be hazardous to users; such systems can be leveraged to report such information to the public. Examining policy elements such as safety provisions, along with others that impact consumer purchase behaviors (e.g., price and taxation of legal vaping products) (Hall et al. 2019; National Academies of Science Engineering and Medicine 2018; Smart and Pacula 2019), remains important areas for further study in the context of EVALI.

Alongside general gaps in knowledge about the health effects of using vaping products (National Academies of Science Engineering and Medicine 2018), it should be noted that the limitations placed on research into cannabis means that there are many unknowns concerning cannabis product safety, particularly for novel products (e.g., vaping products, edibles) (National Academies of Science Engineering and Medicine 2017). Additionally, the heterogeneity of policy elements and level of enforcement across states likely factor into what, if any, level of protection legal environments may actually afford (Hall et al. 2019; Smart and Pacula 2019). It is possible that policy elements that may protect against health issues such as EVALI may also have indirect or unintended adverse public health effects pertinent to other important considerations in the legalization of cannabis use. For instance, legalized policies have been shown to facilitate heavier consumption among heavy cannabis users, which may increase susceptibility to dependence and cannabis use disorder (Hall et al. 2019; National Academies of Science Engineering and Medicine 2017; Smart and Pacula 2019). Further, many legal environments have failed to suppress thriving black markets for cannabis (e.g., California), which consumers may continue to access in spite of legalization due to price, availability, convenience, or other factors (Hall et al. 2019; National Academies of Science Engineering and Medicine 2017; Smart and Pacula 2019). The effect of state actions in response to the outbreak is also worth noting. For example, during the outbreak, the state of Oregon obtained reports of legally obtained cannabis vaping products being a source of EVALI-related contaminants. In turn, the State issued a recommendation for cannabis retailers to review their products for potential safety concerns and post warning signs about potential dangers of using vaping devices (Flaccus 2019). Many states (e.g., Washington, Colorado) implemented bans on the use of vitamin E acetate in vaping products (Boudi et al. 2019), while others still (e.g., Massachusetts) issued complete bans on the use of vaping products for any substance during and following the outbreak (Lannan n.d.). The nature and interaction of specific policy elements implemented by each state, the strength and duration of policy elements, and the impact of black market product availability, consumer behavior, and government actions in response to the EVALI outbreak are all important areas for future research on this topic.

Although this piece represents a novel perspective on potential contributing factors into EVALI, there are limitations to consider. Primarily, we acknowledge that these estimates may be flawed due to the use of information available in the public domain. However, our searches exhibited reasonable concordance with categorical estimates provided by CDC, which lends credibility to the data used for analysis. Additionally, the use of NSDUH data from 2017 to 2018 as a reference population may result in a slight overestimation of cumulative incidence of EVALI, since prevalence of cannabis use has been increasing over time (Hall et al. 2019). Unfortunately, these were the most recent data available; future assessments should aim to replicate these figures with more recent data on the US cannabis user population at the state-level. Case reporting discrepancies between state health departments and the CDC, as well as misreporting of cannabis use by patients (Blount et al. 2019b), may also play a role. Further, there is an absence of national-level data to use to estimate the proportion of cannabis users that have used cannabis oil vaping products (including those obtained through legal or illegal channels). Cannabis can be consumed in many ways, is most often smoked, and is subject to extensive poly-use along with other substances, such as nicotine (National Academies of Science Engineering and Medicine 2017; Werner et al. 2020). Therefore, not all cannabis users to will engage with cannabis oil vaping products, which may further affect our calculations to an unknown degree. Our results may have also been influenced by enactment of state-level executive orders in the wake of the EVALI outbreak. For instance, Massachusetts enacted a ban on the sale of any vaping products shortly after the beginning of the peak of this outbreak (Lannan n.d.). A ban may have exerted a protective effect or drove users to obtain vaping products from the illicit market. While our calculations showed that Massachusetts exhibited similar overall rates of EVALI to places where cannabis was fully illegal, the myriad of contributing and intersecting factors that could potentially drive our findings (e.g., executive order issuance, state-level density of retail dispensaries) remain important areas for future research. Finally, our assumptions and analyses rely on the current evidence base related to EVALI, which is continually emerging. Future assessments should take these factors into account when identifying additional contributors to EVALI.

## Conclusion

Our estimates suggest there may have been a protective effect of state-level, recreational adult-use cannabis policies on incident EVALI. The role of state-level cannabis policy elements, including laboratory testing, product quality standards, and product tracking systems should be considered, along with other geographic indices. These findings may be useful in providing added context to possible contributors into causes of EVALI.

## Data Availability

The datasets used and/or analyzed during the current study are available from the corresponding author upon request. Data obtained from the National Survey on Drug Use and Health (NSDUH) can be found at https://www.samhsa.gov/data/report/2016-2017-nsduh-national-maps-prevalence-estimates-state. Data obtained from the American Community Survey can be found at https://data.census.gov/cedsci/table?q=United%20States&g=0100000US&tid=ACSDP1Y2018.DP05.

## Abbreviations

CDC: US Centers for Disease Control and Prevention
EVALI: E-cigarette or vaping product use–associated lung injury
NSDUH: National Survey on Drug Use and Health
THC: Delta-9-tetrahydrocannabinol
US: United States
